# Supplementary material for: Aerial small target detection algorithm based on cross-scale separated attention
Source: PLoS One. 2025 Nov 26;20(11):e0337318. doi: 10.1371/journal.pone.0337318 (PMC12654883; doi:10.1371/journal.pone.0337318)
Supplement: S1 File — Processed versions (converted to YOLO.txt format) are provided in our GitHub repository (https://github.com/liangjugg/UAS-YOLO/tree/main/datasets). (DOCX) [file pone.0337318.s001.docx]

**S1 File: VisDrone Dataset And Tinyperson Access and Processing Information**

1. Dataset Source

The VisDrone dataset used in this study is publicly available from its official repository maintained by the Tianjin University team: [https://github.com/VisDrone/VisDrone-Dataset](https://github.com/VisDrone/VisDrone-Dataset). We acknowledge the original authors for making this valuable dataset freely available.

2. Dataset Version and Processing

The annotation format was converted from the original .xml (PASCAL VOC) format to .txt (YOLO) format for training compatibility.

3. Access to Processed Dataset

A copy of the processed and formatted dataset used in our experiments is provided in our GitHub repository for the convenience of readers and to ensure the reproducibility of our results: [https://github.com/liangjugg/UAS-YOLO/tree/main/datasets](https://github.com/liangjugg/UAS-YOLO/tree/main/datasets).

4. TinyPerson Dataset

Dataset SourceThe TinyPerson dataset used in this study is publicly available from its official repository maintained by the Institute of Computing Technology, Chinese Academy of Sciences: https://github.com/cleardusk/TinyPerson. We acknowledge the original authors for making this valuable dataset freely available.

Dataset Version and ProcessingFor this research, we utilized the train and val subsets of the TinyPerson dataset. Similar to the VisDrone dataset, the annotation format was converted from the original .json (COCO) format to .txt (YOLO) format for training compatibility.

Access to Processed DatasetA copy of the processed and formatted TinyPerson dataset used in our experiments is also provided in our GitHub repository: https://github.com/liangjugg/UAS-YOLO/tree/main/datasets.
