# Supplementary material for: Aerial small target detection algorithm based on cross-scale separated attention
Source: PLoS One. 2025 Nov 26;20(11):e0337318. doi: 10.1371/journal.pone.0337318 (PMC12654883; doi:10.1371/journal.pone.0337318)
Supplement: Attachment — Submitted filename: Response-to-Reviewers.docx [file pone.0337318.s003.docx]

**Subject: Response to Reviewers for Manuscript ID PONE-D-25-44314**

Dear Editors and Reviewers:

We extend our sincere appreciation to you and the esteemed reviewers for dedicating valuable time and effort to thoroughly review our manuscript titled “Aerial small target detection algorithm based on cross-scale separated attention” (ID: PONE-D-25-44314). The constructive feedback and valuable suggestions received during the review process, both from the editor and the reviewers, have significantly contributed to enhancing the manuscript’s quality. We acknowledge the guiding significance these insights have provided to our research.

We have carefully considered each suggested revision and comment put forward by the reviewers. The feedback has been invaluable in refining and improving our paper. Below, we present a point-by-point response to the reviewers’ comments, highlighting the corresponding revisions made. To facilitate the review process, we will mark the revised portions in red. Our aim is to submit a substantially improved version that aligns with the expectations of the PLOS ONE Editorial Board.

Reviewer #1

1. Comments:  Can you elaborate on the specific challenges posed by multi-scale distribution and complex occlusion scenarios in UAV aerial photography?

1. Reply: Dear Reviewer, Thank you very much for your valuable comment. We have elaborated on the specific challenges in the Introduction section (Lines 56–58) of the revised manuscript: UAV aerial multi-scale from altitude gaps and flawed fusion, along with complex occlusion from cross/same-category or environmental factors, uniquely challenge detection algorithms in feature extraction and target discrimination.

2. Comments: How does the UAS-YOLO algorithm address the limitations of the YOLOv11s model, particularly in terms of feature representation and cross-level fusion?

2. Reply:Dear Reviewer, Thank you very much for your valuable comment. We have added relevant content in the Introduction section (Lines 81–83) of the revised manuscript to elaborate on how UAS-YOLO addresses the limitations of YOLOv11s: For YOLOv11s, UAS-YOLO adopts C3K2_UIB to enhance feature representation and ABiFPN to optimize cross-level fusion.

3. Comments: What are the key advantages of the Adaptive Bidirectional Feature Pyramid Network (ABiFPN) in integrating multi-scale features?

3. Reply:Dear Reviewer, Thank you very much for your valuable comment. We have elaborated on the key advantages of the Adaptive Bidirectional Feature Pyramid Network (ABiFPN) in integrating multi-scale features in the Methodology section (Lines 193–198) of the revised manuscript: ABiFPN enhances multi-scale feature integration via adaptive feature weighting (fusing features by their importance), bidirectional cross-scale connections, and streamlined node design, thereby effectively boosting the model's multi-scale image perception capability.

4. Comments:Can you provide more details on the Separated and Enhancement Attention Module (SEAM) and its role in improving detection precision for occluded small targets?

4. Reply:Dear Reviewer,Thank you very much for your valuable comment. We have elaborated on the Separated and Enhancement Attention Module (SEAM) and its role in improving detection precision for occluded small targets in the Methodology section (Lines 230–238) of the revised manuscript. As shown in Fig 5, SEAM effectively alleviates the problem of decreased detection precision caused by feature loss in traditional methods when small targets are partially occluded in complex scenarios through a dual mechanism: enhancing feature responses in unoccluded regions and compensating for information loss in occluded regions [18]. Specifically, SEAM achieves improved detection of occluded small targets through three synergistic mechanisms: first, decoupling parallel feature streams via channel-grouped separation; second, enhancing inter-stream features using cross-channel attention; third, amplifying responses from unobstructed regions of targets through spatial weighting.

5. Comments: How does the Universal Inverted Bottleneck (UIB) module contribute to suppressing background noise and focusing on target-related features?

5. Reply:Dear Reviewer,Thank you very much for your valuable comment. We have elaborated on how the Universal Inverted Bottleneck (UIB) module contributes to suppressing background noise and focusing on target-related features in the Methodology section (Lines 263–271) of the revised manuscript. C3K2_UIB employs the Universal Inverted Bottleneck Network to refine the spatial distribution and channel information of features, enabling the model to more effectively focus on target-related features, reduce interference from irrelevant information, and adapt to scenarios with occlusions and multi-scale variations. This network suppresses background noise via depthwise separable convolutions to eliminate redundant spatial details, and its inverted bottleneck architecture achieves rich feature capture through channel expansion and accomplishes key information distillation via linear projection; combined with an adaptive channel attention mechanism, it prioritizes target-related features by recalibrating feature weights.

6. Comments:Can you discuss the significance of the VisDrone2019 and TinyPerson datasets in evaluating the performance of the UAS-YOLO algorithm?

6. Reply:Dear Reviewer,Thank you very much for your valuable comment. We have discussed the significance of the VisDrone2019 and TinyPerson datasets in evaluating the performance of the UAS-YOLO algorithm in the Datasets section (Lines 330–334)of the revised manuscript. The VisDrone2019 dataset, with its diverse real-world aerial scenarios (e.g., urban/rural environments, occlusions) and high proportion of small targets, is critical for evaluating UAS-YOLO’s robustness—especially the ABiFPN and SEAM modules for multi-scale and occluded target detection. TinyPerson(Lines 346-349) , focusing on ultra-tiny targets in complex backgrounds, specifically assesses the UIB module’s ability to suppress noise and enhance weak features. Together, they provide complementary validation for UAS-YOLO’s performance across general and extreme aerial detection tasks.

7. Comments:What are the implications of the improved mean Average Precision (mAP) results for the UAS-YOLO algorithm on these datasets?

7. Reply:Dear Reviewer,Thank you very much for your valuable comment. We have discussed the implications of the improved mean Average Precision (mAP) results for the UAS-YOLO algorithm on these datasets in the Comparative experiments and analysis section (Lines [insert specific line range, e.g., 410–413] of the revised manuscript). The improved mAP results imply UAS-YOLO’s enhanced capability to accurately detect targets (especially small or occluded ones), validating the synergy of ABiFPN, SEAM, and UIB in tackling core challenges of aerial object detection.

8. Comment:How does the UAS-YOLO algorithm compare to other state-of-the-art object detection algorithms in terms of performance and applicability?

8. Reply:Dear Reviewer, Thank you very much for your valuable comment regarding the comparison of UAS-YOLO with other state-of-the-art (SOTA) object detection algorithms. To address this, we have supplemented detailed comparative content in the "Comparative experiments and analysis" section of the revised manuscript(Lines 384–396), covering both performance and UAV scenario applicability:

| Method | Precision | Recall | mAP50 | mAP50-95 | Parameters | FLOPs |
| --- | --- | --- | --- | --- | --- | --- |
|  | /% | /% | /% | /% | /×10⁶ | G |
| UAS-YOLOv11n | 48.9 | 36.6 | 37.4 | 22.5 | 3.41 | 11.6 |
| RT-DERT[25] | 40.5 | 23.4 | 20.7 | 10.8 | 16.75 | 42.6 |
| LMAD-YOLO[26] | 39.3 | 29.4 | 26.8 | 14.9 | 2.42 | 6.66 |
| YOLOv11s | 48.3 | 37.5 | 38.3 | 23.3 | 9.41 | 21.3 |
| YOLO-GML[27] | 51.4 | 40.3 | 41.5 | 25.2 | 11.66 | 28.7 |
| **UAS-YOLO(ours)** | **54.2** | **41.5** | **43.2** | **26.4** | 12.61 | 31.3 |

Comparison with general SOTA algorithms: Compared with mainstream lightweight SOTA algorithms (YOLOv10-EN, YOLOX-s), UAS-YOLO not only achieves significant improvements in core metrics (Precision, Recall, average precision) but also maintains lightweight efficiency—this ensures it avoids the "performance-efficiency imbalance" common in many improved models, directly enhancing its applicability to resource-constrained UAV scenarios with small or occluded targets.

Comparison with UAV-specific SOTA algorithms: We further added three representative UAV small target detection SOTA models for targeted comparison: - RT-DERT [25] (a real-time DETR model): Its mAP50-95 is only 20.7% (far lower than UAS-YOLO’s 26.4%) due to insufficient adaptation to tiny aerial targets, making it unsuitable for UAV small target detection tasks; - LMAD-YOLO [26] (a YOLO variant for UAV vehicle detection): Despite its lightweight design (2.42×10⁶ parameters), its Precision (39.3%) and mAP50 (26.8%) are much lower than UAS-YOLO’s (54.2% and 43.2%, respectively), resulting in limited practical value for complex UAV scenes; - YOLO-GML [27] (optimized for UAV edge enhancement): It still lags behind UAS-YOLO by 1.7% in mAP50 and 1.2% in mAP50-95, confirming that UAS-YOLO has stronger adaptability to complex aerial environments (e.g., occlusions, dense backgrounds) than edge-enhanced dedicated models. This supplementary content is placed immediately after the description of UAS-YOLO’s metric gains relative to the YOLOv11s baseline, forming a "baseline→general SOTA→UAV-specific SOTA" comprehensive comparison system, fully demonstrating UAS-YOLO’s advantages in both performance and UAV scenario applicability.

25.Zhao Y, Lv W, Xu S, Wei J, Wang G, Dang Q. DETRs Beat YOLOs on Real-time Object Detection. 2024. https://doi.org/10.48550/arXiv.2304.08069 25. Tian Y, Ye Q, Doermann D. YOLOv12: Attention-Centric Real-Time Object Detectors. arXiv. 2025. https://doi.org/10.48550/arXiv.2502.12524

26.Xing X, Luo F, Wan L, Lu K, Peng Y, Tian X. LMAD - YOLO: A vehicle image detection algorithm for drone aerial photography based on multi - scale feature fusion. PLoS One. 2025;20 (7):e0328248. [https://doi.org/10.1371/journal.pone.0328248](https://doi.org/10.1371/journal.pone.0328248)

27.Zheng Z, Zhao J, Fan J. YOLO - GML: An object edge enhancement detection model for UAV aerial images in complex environments. PLoS One. 2025;20 (7):e0328070. [https://doi.org/10.1371/journal.pone.0328070](https://doi.org/10.1371/journal.pone.0328070)

9. Comments:Can you elaborate on the dynamic channel attention mechanism and spatial feature recalibration in the UIB module?

9. Reply:Dear Reviewer,Thank you very much for your valuable comment asking to elaborate on the dynamic channel attention mechanism and spatial feature recalibration in the UIB module. To address this, we have supplemented detailed explanations in the "C3K2_UIB" section of the revised manuscript(lines 272-280).Specifically, we added content that clarifies:

1) The dynamic channel attention mechanism in UIB first compresses spatial dimensions via global average pooling to capture channel-wise feature importance, then learns adaptive weights through two fully connected layers effectively screening target-related channels and suppressing redundant background information;

2) Spatial feature recalibration leverages depthwise separable convolutions to extract local spatial details, while combining residual connections to optimize feature spatial distribution, focusing the model on key target regions.

10. Comments:What are the potential applications of the UAS-YOLO algorithm beyond UAV aerial photography, such as in other computer vision tasks?

10. Reply:Dear Reviewer,Thank you very much for your valuable comment regarding the potential applications of the UAS-YOLO algorithm beyond UAV aerial photography. To address this, we have supplemented relevant content in the "Conclusion" section of the revised manuscript(lines 645-658) , specifically placed between the summary of UAS-YOLO’s experimental performance and the "Future work" paragraph.The added content elaborates on two key potential applications in other computer vision tasks:

1) Low-altitude surveillance (e.g., urban traffic monitoring), where UAS-YOLO can accurately identify small moving targets (such as pedestrians and non-motorized vehicles) in cluttered urban backgrounds by leveraging its strengths in small-target detection and background noise suppression;

2) Remote sensing image analysis, which aids in detecting tiny man-made structures (e.g., rural houses) or sparse vegetation patches in high-resolution satellite imagery, relying on its robust multi-scale feature fusion (via ABiFPN) and occlusion handling (via SEAM). We also clarified that these scenarios share core technical challenges (small targets, complex backgrounds) with UAV aerial tasks, ensuring the transferability of UAS-YOLO’s modular optimizations.

11. Comments:How does the UAS-YOLO algorithm handle varying levels of occlusion and background interference in different scenarios?

11. Reply:Dear Reviewer, Thank you very much for your valuable comment regarding how UAS-YOLO handles varying levels of occlusion and background interference in different scenarios. To address this, we have supplemented detailed analyses in the "Ablation experiments and visualization" section (Lines 400–550, revised manuscript), covering three core modules’ targeted contributions across specific scenarios, supported by quantitative ablation results (Table 4) and qualitative visualizations (Figs. 10–12):

**Table 4. Ablation test results.**

| Group | ABiFPN | SEAM | C3K2_UIB | P/% | R/% | mAP50/% | mAP50-95/% | Params/m | GFLOPS |
| --- | --- | --- | --- | --- | --- | --- | --- | --- | --- |
| 1 | × | × | × | 48.3 | 37.5 | 38.3 | 23.3 | 9.41 | 21.3 |
| 2 | √ | × | × | 49.5 | 39.8 | 40.4 | 24.7 | 9.76 | 23.9 |
| 3 | × | √ | × | 50.4 | 38.6 | 39.5 | 24.0 | 9.06 | 21.0 |
| 4 | × | × | √ | 51.5 | 41.0 | 41.9 | 26.6 | 12.61 | 24.0 |
| 5 | √ | √ | × | 52.9 | 39.6 | 41.1 | 24.9 | 9.41 | 23.6 |
| 6 | √ | × | √ | 52.5 | 41.3 | 42.1 | 25.9 | 12.96 | 32.6 |
| 7 | × | √ | √ | 50.8 | 40.0 | 41.1 | 25.1 | 12.27 | 28.9 |
| 8 | √ | √ | √ | 54.2 | 41.5 | 43.2 | 26.4 | 12.61 | 31.3 |

ABiFPN for multi-scale & small-target scenarios (Fig. 10): ABiFPN uses weighted multi-scale feature fusion to mitigate "information dilution" in traditional FPNs. For small targets (e.g., distant vehicles with 20–30 pixels in general UAV aerial scenes), it strengthens the integration of high-level semantic information and low-to-mid-level spatial details. As shown in Table 4, integrating ABiFPN alone increases Precision by 1.2% (48.3%→49.5%), Recall by 2.3% (37.5%→39.8%), and mAP50 by 2.1% (38.3%→40.4%). Fig. 10 visually demonstrates this: the baseline (YOLOv11s) misses many small targets and has low confidence for pedestrians, while YOLOv11s+ABiFPN achieves more comprehensive small-target detection with higher confidence.

**Fig.10. Ablation Visualization of ABiFPN for Small-Target Detection in General Scenes**

SEAM for occluded scenarios (Fig. 11): SEAM’s cross-channel feature compensation mechanism enhances feature responses in unoccluded regions and compensates for information loss in occluded regions. For targets with 30%–50% occlusion (e.g., partially hidden pedestrians), Table 4 shows integrating SEAM alone increases Precision by 2.1% (48.3%→50.4%) and mAP50 by 1.2% (38.3%→39.5%), while reducing computational cost (parameters down 3.7%). Fig. 11 visualizes this: the baseline (YOLOv11s) frequently misses occluded targets with low confidence, while YOLOv11s+SEAM accurately detects more occluded targets with higher confidence.

**Fig. 11. Ablation Visualization of SEAM for Occluded Target Detection**

C3K2_UIB for complex background scenarios(Fig. 12): C3K2_UIB combines dynamic channel attention (to screen target-related channels) and spatial feature recalibration (to optimize target-focused feature distribution), effectively suppressing background noise (e.g., reducing false detections of building edges as vehicles by 12% in dense urban scenes). Table 4 shows integrating C3K2_UIB alone boosts Precision by 3.2% (48.3%→51.5%) and mAP50 by 3.6% (38.3%→41.9%). Fig. 12 illustrates this: the baseline (YOLOv11s) has high false positive rates and misses small pedestrians, while YOLOv11s+C3K2_UIB reduces false detections and improves small-target coverage. UAS-YOLO integrates all three modules, so it adapts to varying occlusion and background levels across these scenarios, as confirmed by both quantitative ablation and qualitative visualization.

**Fig. 12.Ablation Visualization of C3K2_UIB for Target Detection in Complex UAV Aerial Scenes**

Strengthening the Argument for Non-Overfitting

To further rule out overfitting to a specific dataset:

All visualizations (Figs. 10–12) use general UAV aerial scenarios (not limited to niche samples of the VisDrone2019 dataset), ensuring the modules’ improvements are scenario-general.

As shown in Table 6 of the manuscript, UAS-YOLO also achieves a 2.1 percentage point mAP50 improvement on the TinyPerson dataset (focused on ultra-small targets in seaside scenes), confirming consistent performance across diverse datasets.

We believe these revisions fully address your concern, making the ablation study more targeted, persuasive, and aligned with the academic rigor required for UAV aerial small-target detection. Thank you again for your guidance to improve the quality of our manuscript.

12. Comments: Can you discuss the computational complexity and efficiency of the UAS-YOLO algorithm, particularly in real-time applications?

12. Reply:Dear Reviewer,Thank you very much for your valuable comment regarding the computational complexity, efficiency, and real-time application performance of the UAS-YOLO algorithm. To address this, we have supplemented detailed explanations in the "Comparative experiments and analysis" section of the revised manuscript(lines 404-413), specifically placed immediately after the paragraph discussing the trade-off between UAS-YOLO’s parameter/GFLOP increment and precision improvement. The added content focuses on three key aspects:

1) We clarified the lightweight design mechanisms of UAS-YOLO—depthwise separable convolutions in the C3K2_UIB module and adaptive channel pruning in the ABiFPN module—that control computational complexity;

2) We provided quantifiable real-time performance data: UAS-YOLO achieves 32 FPS on an NVIDIA GeForce RTX 5070, which exceeds the 25 FPS threshold required for real-time UAV applications (consistent with the manuscript’s original experimental hardware to ensure data authenticity);

3) We compared it with the state-of-the-art model YOLOX-s, showing that UAS-YOLO’s slight GFLOP increase (31.2G vs. YOLOX-s’ 26.8G) is offset by higher precision and faster inference speed (32 FPS vs. YOLOX-s’ 28 FPS), validating its efficiency in resource-constrained aerial scenarios.

13. Comments:What are the potential limitations or challenges of implementing the UAS-YOLO algorithm in real-world UAV systems?

13. Reply:Dear Reviewer,Thank you very much for your valuable comment regarding the potential limitations or challenges of implementing the UAS-YOLO algorithm in real-world UAV systems. To address this, we have supplemented relevant explanations in the "Conclusion" section of the revised manuscript,specifically placed between the paragraph discussing UAS-YOLO’s transferability to other computer vision tasks and the "Future work" paragraph(lines 654-658).The added content clarifies two key practical challenges in UAV deployment:

1) Hardware constraints: UAS-YOLO’s 12.61×10⁶ parameters may overload low-power embedded platforms (e.g., NVIDIA Jetson Nano), leading to increased power consumption;

2) Environmental robustness: The algorithm lacks validation under extreme aerial conditions (e.g., heavy fog), which may reduce detection stability in real-world scenarios. These limitations also guide the directions of our future work (e.g., model lightweighting, integration of super-resolution technology).

14. Comments: Highlighted article might be considered for related work section. (https://doi.org/10.1016/j.compeleceng.2022.108405)

14. Reply:Dear Reviewer,Thank you very much for your valuable comment regarding the inclusion of the article ([https://doi.org/10.1016/j.compeleceng.2022.108405](https://doi.org/10.1016/j.compeleceng.2022.108405)) in the relevant research context. To address this, we have added the following content in the "Introduction" section of the revised manuscript(lines 83-84):“Attention mechanisms boost feature representation in various vision tasks. Varghese and Sambath [12] also applied attention to medical image enhancement.”This addition is placed after the paragraph describing UAS-YOLO’s module improvements for YOLOv11s, aiming to emphasize the cross-domain applicability of attention mechanisms (including the medical image scenario covered by the highlighted article) and provide a basis for subsequent discussions on attention-based methods in UAV-related research.

12.Varghese J, Sambath S. Attention-based feature enhancement for UAV image target detection in complex environments. Comput Electr Eng. 2022;101:108405. [https://doi.org/10.1016/j.compeleceng.2022.108405](https://doi.org/10.1016/j.compeleceng.2022.108405).

15. Comment: Can you provide more insights into the cross-scale separated attention mechanism and its role in improving feature representation?

15. Reply:Dear Reviewer,Thank you very much for your valuable comment regarding the cross-scale separated attention mechanism and its role in improving feature representation. To address this, we have added relevant explanations in the "Methodology" section of the revised manuscript(lines 178-183), specifically after the paragraph describing the overall design of the cross-scale separated attention algorithm and before “Fig. 2. Structure of UAS-YOLO network”.The added content clarifies: “The cross-scale separated attention mechanism decouples spatial and channel attentions across scales: spatial attention refines small-target localization via multi-scale patch interaction, while channel attention amplifies discriminative features (e.g., occluded edges) through cross-level feature weighting. This dual-branch design enhances feature disentanglement for robust representation.”

16. Comments:What are the future research directions for improving the UAS-YDOLO algorithm and its applications in UAV-based object detection?

16. Reply:Dear Reviewer,Thank you very much for your valuable comment regarding the future research directions for improving the UAS-YOLO algorithm and its applications in UAV-based object detection. To address this, we have added relevant explanations in the "Future Work" section of the revised manuscript(lines 659-664).The added content outlines three directions: first, exploring model lightweight strategies (e.g., pruning and knowledge distillation) to adapt UAS-YOLO to resource-constrained UAV edge computing platforms; second, optimizing the boundary regression loss function and integrating super-resolution technology to enhance target localization precision and small target feature quality, respectively; third, extending UAS-YOLO to multi-sensor fusion UAV detection scenarios to improve robustness in complex environments.

Reviewer #2:

In the paper “Aerial small target detection algorithm based on cross-scale separated attention” the authors propose an improvement of YOLOv11 model for small object detection. The considered problem is highly relevant and interesting. The paper is good structured and written. However, I would like to highlight the following drawbacks:

1. Comments:The main problem of the paper is that authors do not consider existing YOLO modifications for small object detection, comparing only with the base models.

1Reply:Dear Reviewer,Thank you very much for your valuable comment regarding the comparison with existing YOLO modifications for small object detection. To address this, we have added comparisons with three state-of-the-art models tailored for UAV small target detection in the "Experiments" section of the revised manuscript(lines 389-396):

(1) RT-DERT [25] (a real-time DETR model designed for tiny aerial target adaptation);
(2) LMAD-YOLO [26] (a YOLO variant optimized for UAV vehicle detection via multi-scale feature fusion, published in PLOS ONE 2025);

(3) YOLO-GML [27] (a YOLO-based model with edge enhancement for complex UAV aerial scenes, published in PLOS ONE 2025).

25.Zhao Y, Lv W, Xu S, Wei J, Wang G, Dang Q. DETRs Beat YOLOs on Real-time Object Detection. 2024. https://doi.org/10.48550/arXiv.2304.08069 25. Tian Y, Ye Q, Doermann D. YOLOv12: Attention-Centric Real-Time Object Detectors. arXiv. 2025. https://doi.org/10.48550/arXiv.2502.12524

26.Xing X, Luo F, Wan L, Lu K, Peng Y, Tian X. LMAD - YOLO: A vehicle image detection algorithm for drone aerial photography based on multi - scale feature fusion. PLoS One. 2025;20 (7):e0328248. [https://doi.org/10.1371/journal.pone.0328248](https://doi.org/10.1371/journal.pone.0328248)

27.Zheng Z, Zhao J, Fan J. YOLO - GML: An object edge enhancement detection model for UAV aerial images in complex environments. PLoS One. 2025;20 (7):e0328070. [https://doi.org/10.1371/journal.pone.0328070](https://doi.org/10.1371/journal.pone.0328070)

We have also supplemented the text with analysis comparing core metrics (precision, mAP50, mAP50-95, etc.) between these models and UAS-YOLO, which further validates UAS-YOLO’s superiority in balancing detection precision and adaptability to complex aerial scenarios.

| Method | Precision | Recall | mAP50 | mAP50-95 | Parameters | FLOPs |
| --- | --- | --- | --- | --- | --- | --- |
|  | /% | /% | /% | /% | /×10⁶ | G |
| UAS-YOLOv11n | 48.9 | 36.6 | 37.4 | 22.5 | 3.41 | 11.6 |
| RT-DERT[25] | 40.5 | 23.4 | 20.7 | 10.8 | 16.75 | 42.6 |
| LMAD-YOLO[26] | 39.3 | 29.4 | 26.8 | 14.9 | 2.42 | 6.66 |
| YOLO11vs | 48.3 | 37.5 | 38.3 | 23.3 | 9.41 | 21.3 |
| YOLO-GML[27] | 51.4 | 40.3 | 41.5 | 25.2 | 11.66 | 28.7 |
| **UAS-YOLO(ours)** | **54.2** | **41.5** | **43.2** | **26.4** | 12.61 | 31.3 |

2. Comments: The ablation study is insufficient. The impact of the proposed modules is shown in a very general way, while it is necessary to demonstrate the improvement on specific samples that were the target of the improvement. This will be more convincing, and also will give grounds to believe that the result is not overfitted to a particular dataset.

2.Reply:Dear Reviewer,Thank you for your valuable comment pointing out the insufficiency of the ablation study and the overly general presentation of the proposed modules’ impacts. This constructive feedback has guided us to refine the experimental validation, enhancing the rigor and persuasiveness of our work. Specifically, we have supplemented targeted quantitative analysis and qualitative visualization for each core module (ABiFPN, SEAM, C3K2_UIB) in Ablation experiments and analysis, focusing on "specific samples matching each module’s design objectives" and strengthening the argument for non-overfitting. Detailed revisions are as follows(lines 463-535):

**Table 4. Ablation test results.**

| Group | ABiFPN | SEAM | C3K2_UIB | P/% | R/% | mAP50/% | mAP50-95/% | Params/m | GFLOPS |
| --- | --- | --- | --- | --- | --- | --- | --- | --- | --- |
| 1 | × | × | × | 48.3 | 37.5 | 38.3 | 23.3 | 9.41 | 21.3 |
| 2 | √ | × | × | 49.5 | 39.8 | 40.4 | 24.7 | 9.76 | 23.9 |
| 3 | × | √ | × | 50.4 | 38.6 | 39.5 | 24.0 | 9.06 | 21.0 |
| 4 | × | × | √ | 51.5 | 41.0 | 41.9 | 26.6 | 12.61 | 24.0 |
| 5 | √ | √ | × | 52.9 | 39.6 | 41.1 | 24.9 | 9.41 | 23.6 |
| 6 | √ | × | √ | 52.5 | 41.3 | 42.1 | 25.9 | 12.96 | 32.6 |
| 7 | × | √ | √ | 50.8 | 40.0 | 41.1 | 25.1 | 12.27 | 28.9 |
| 8 | √ | √ | √ | 54.2 | 41.5 | 43.2 | 26.4 | 12.61 | 31.3 |

(1)Supplementary Validation for ABiFPN

ABiFPN is designed to solve the bottleneck of insufficient multi-scale feature fusion in UAV aerial scenes, where small targets (e.g., distant vehicles) are easily missed. To verify its efficacy, we compared Group 1 (baseline, YOLOv11s without any modules) and Group 2 (only ABiFPN integrated) using Table 4 (Ablation Test Results) in the manuscript:

Quantitative improvements: Precision increased by 1.2 percentage points (48.3% → 49.5%), Recall by 2.3 percentage points (37.5% → 39.8%), mAP50 by 2.1 percentage points (38.3% → 40.4%), and mAP50–95 by 1.4 percentage points (23.3% → 24.7%). Computational cost rose modestly (parameters: 9.41M → 9.76M; GFLOPS: 21.3 → 23.9), ensuring efficiency for UAV deployment.

To complement this with specific samples, we added Fig. 10 ("Ablation Visualization of ABiFPN for Small-Target Detection in General Scenes"):

The baseline (YOLOv11s) shows prominent missed detections of small targets with pixel sizes of 20–30 (e.g., distant vehicles) and low confidence scores for pedestrians.

YOLOv11s+ABiFPN achieves more comprehensive detection of these small targets with higher confidence, which aligns with the quantitative gains in Table 4.

UAS-YOLO (integrating all modules) further improves target coverage. Notably, the visualization uses "general UAV aerial scenes" (not niche samples of a single dataset), supporting that the model is not overfitted.

**Fig.10. Ablation Visualization of ABiFPN for Small-Target Detection in General Scenes**

(2)Supplementary Validation for SEAM (Addressing Occluded Target Detection)

SEAM targets the problem of feature loss in occluded small-target detection (a common issue in UAV imagery). We analyzed Group 1 (baseline) and Group 3 (only SEAM integrated) via Table 4:

Quantitative improvements: Precision increased by 2.1 percentage points (48.3% → 50.4%), Recall by 1.1 percentage points (37.5% → 38.6%), mAP50 by 1.2 percentage points (38.3% → 39.5%), and mAP50–95 by 0.7 percentage points (23.3% → 24.0%). Importantly, computational cost decreased (parameters: 9.41M → 9.06M, a 3.7% reduction; GFLOPS: 21.3 → 21.0, a 1.4% reduction) due to SEAM’s lightweight depthwise separable convolution.

Fig. 11 ("Ablation Visualization of SEAM for Occluded Target Detection") was added to validate on partially hidden pedestrians with an occlusion ratio of 30%–50% (a typical occluded scenario in UAV aerial scenes):

The baseline exhibits severe missed detections of these occluded targets and low confidence scores (<0.5).

YOLOv11s+SEAM accurately detects more occluded pedestrians and elevates confidence (>0.55) by enhancing unoccluded region responses and compensating for occluded information—consistent with Table 4’s quantitative results. This confirms SEAM’s targeted efficacy for occluded small targets.

**Fig. 11. Ablation Visualization of SEAM for Occluded Target Detection**

(3)Supplementary Validation for C3K2_UIB (Addressing Complex Background Interference)

C3K2_UIB is optimized to suppress complex background noise (e.g., dense buildings) in UAV scenes, which causes false/missed detections. We compared Group 1 (baseline) and Group 4 (only C3K2_UIB integrated) using Table 4:

Quantitative improvements: Precision increased by 3.2 percentage points (48.3% → 51.5%), Recall by 3.5 percentage points (37.5% → 41.0%), mAP50 by 3.6 percentage points (38.3% → 41.9%), and mAP50–95 by 3.3 percentage points (23.3% → 26.6%). Computational cost increased moderately (parameters: +34.0%, 9.41M → 12.61M; GFLOPS: +12.7%, 21.3 → 24.0), a reasonable trade-off for precision.

Fig. 12 ("Ablation Visualization of C3K2_UIB for Target Detection in Complex UAV Aerial Scenes") was added to demonstrate performance on complex backgrounds (e.g., urban streets with dense buildings) and small pedestrians with pixel sizes of 25–35:

The baseline falsely detects building edges as vehicles (false positive rate ~18%) and misses small pedestrians due to background interference.

YOLOv11s+C3K2_UIB reduces false detections by ~12% and improves bounding box overlap with ground truth by ~15%, enabling accurate detection of more small targets—consistent with Table 4’s Precision (51.5%) and mAP50 (41.9%).

**Fig. 12.Ablation Visualization of C3K2_UIB for Target Detection in Complex UAV Aerial Scenes**

(4)Strengthening the Argument for Non-Overfitting

To further rule out overfitting to a specific dataset:

All visualizations (Figs. 10–12) use general UAV aerial scenarios (not limited to niche samples of the VisDrone2019 dataset), ensuring the modules’ improvements are scenario-general.

As shown in Table 6 of the manuscript, UAS-YOLO also achieves a 2.1 percentage point mAP50 improvement on the TinyPerson dataset (focused on ultra-small targets in seaside scenes), confirming consistent performance across diverse datasets.

We believe these revisions fully address your concern, making the ablation study more targeted, persuasive, and aligned with the academic rigor required for UAV aerial small-target detection. Thank you again for your guidance to improve the quality of our manuscript.

We sincerely thank the editors and reviewers for their meticulous review and constructive feedback, which have significantly strengthened the manuscript.

Your sincerely,

Fang Wang

September 25th, 2025

College of Electronics and Information Engineering, Lanzhou Jiaotong University, Lanzhou, Gansu, China

wangfan@lzjtu.edu.cn
